# Autonomic regulation in muscular dystrophy

**DOI:** 10.3389/fphys.2014.00061

**Published:** 2014-02-17

**Authors:** Rasna Sabharwal

**Affiliations:** Department of Internal Medicine, University of Iowa Carver College of MedicineIowa City, IA, USA

**Keywords:** muscular dystrophy, sympathetic nervous system, parasympathetic nervous system, cardiomyopathy

Muscular dystrophies are a heterogeneous group of genetic muscle diseases characterized by progressive muscle weakness and atrophy. Cardiomyopathy and congestive heart failure or conduction system abnormalities that cause arrhythmias, and sudden cardiac death have been observed in muscular dystrophies. Despite the extensive research and ongoing clinical trials in muscular dystrophy to improve skeletal muscle pathology and function, there is no effective treatment for this devastating disease. Existence of an association between lethal arrhythmias and signs of autonomic imbalance has initiated efforts to determine quantitative markers of autonomic activity in neuromuscular diseases. To what extent unabated sympathetic overdrive contributes to the eventual demise of skeletal and cardiac muscle remains to be determined. These unexplored questions led us to compile this research topic entitled “Autonomic Regulation in Muscular Dystrophy” to help improve our understanding of the role of autonomic nervous system in muscular dystrophy.

Angelini et al. in their overview have compiled together evidence of aberrant autonomic signaling in a variety of clinically defined muscular dystrophies (Angelini et al., 2013). Duchenne (DMD) and Becker (BMD) muscular dystrophies are X-linked recessive disorders due to complete or partial loss of the dystrophin protein. Tachycardia, atrial and ventricular arrhythmias, sweating, and reduced heart rate variability (HRV) are common symptoms seen in DMD and BMD patients, even in the absence of overt cardiomyopathy. These autonomic changes were present early in the course of DMD/BMD and became more marked with the progression of the disorder. In myotonic dystrophy 1 (MD1), caused by (CTG)n expansion in the gene encoding dytrophia-myotonica-protein kinase (DMPK), orthostatic hypotension, heart rhythm conduction disturbances, abnormal gut movements, reduced HRV, and disorders of sweating are relatively common. In their review, Angelini et al. have described that increased sympathetic activity and an abnormal vasoconstrictor response are evident in other forms of muscular dystrophy including Facio Scapulo Humeral muscular dystrophy (FSHD), Emery-Dreifuss muscular dystrophy (EDMD), and Miyoshi muscular dystrophy (MMD). In Fukuyama-type congenital muscular dystrophy (FCMD), decreased numbers of catecholaminergic neurons in cardiovascular and respiratory centers of the brain has been suggested as a cause of sudden death.

Whilst increased sympathetic activity and reduced parasympathetic activity is now recognized as a hallmark of muscular dystrophy, the mechanisms that contribute to this imbalance are poorly understood. Smith and colleagues in their review discuss the possibility of the exercise pressor reflex as one such mechanism that contributes to the autonomic imbalance in muscular dystrophy. In muscular dystrophy, this reflex is possibly exaggerated to cause an extreme activation of the sympathetic nervous system during mild/moderate exercise. Exaggerated sympathetic activation could restrain blood flow, arteriolar dilatation, and capillary recruitment leading to under perfused areas of the working dystrophic muscles. Exaggerated sympathetic activation would eventually increase the risk of developing myocardial ischemia and cardiac arrest in muscular dystrophy patients (Smith et al., 2014).

Dystrophin deficiency in DMD/BMD causes loss of muscle-specific isoform of neuronal nitric oxide synthase (nNOSμ) to reduce signaling of muscle-derived nitric oxide (NO). Normally, during muscle contraction nNOSμ-derived NO diffuses to the nearby microvessels to increase cGMP and reduce norephineprine-mediated vasoconstriction. However, in DMD/BMD lack of nNOSμ-derived NO causes unrestrained sympathetic vasoconstriction and functional muscle ischemia during exercise. As a consequence, the diseased muscles are prone to injury during exercise in DMD/BMD. Strategies to improve nNOSμ-NO signaling in dystrophic muscles as a promising therapeutic intervention are discussed by Thomas (2013).

Review by Rudolf et al. provides evidence of altered cAMP-signaling in skeletal muscles of humans and *mdx* mice with muscular dystrophy. In the skeletal muscles, catecholamines primarily stimulate β_2_-adrenergic receptors to regulate physiological functions such as blood flow and metabolism. β_2_-adrenergic receptors are G-protein-coupled receptors that couple to Gαs to activate adenyl cyclase leading to an increase in cAMP. The distribution of β_2_-adrenergic receptors is severely disturbed in the dystrophic skeletal muscle. Therefore, improving the cAMP-signaling in muscular dystrophy might be an effective approach that needs to be investigated further (Rudolf et al., 2013).

Lastly, in the article by Sabharwal (2014), the link between psychological stress disorders and autonomic dysfunction in muscular dystrophy has been speculated. Due to social and physical hardships patients with muscular dystrophy e.g., MD1, DMD, and limb girdle muscular dystrophy (LGMD) may exhibit symptoms of psychological stress disorders such as anxiety and depression. Psychological stress is an independent predictor of mortality. In addition, there exists strong co-morbidity between cardiovascular risk and stress disorders, such that presence of one increases the likelihood of developing the other. Thus, it is logical to speculate that autonomic dysfunction and psychological stress synergistically exacerbate progression of muscular dystrophy, and the potential mechanisms involved have been discussed. The renin angiotensin system (RAS) plays a vital role in cardiovascular homeostasis. RAS comprises of two axis namely, angiotensin II (Ang II) that acts via type 1 and type 2 receptors, and angiotensin-(1–7) acting via Mas receptors. While the sympathoexcitatory, profibrotic, vasoconstrictor, and stress-inducing effects of Ang II are widely recognized in many diseases, it is now becoming evident that Ang-(1–7) is an important peptide that can counter-regulate these actions. I have discussed how RAS peptides and selective serotonin reuptake inhibitors can improve autonomic function and reduce psychological stress in muscular dystrophy.

The articles included in this research topic highlight mechanisms that contribute to autonomic dysfunction in muscular dystrophy. Furthermore, these articles will not only stimulate further research but also encourage use of new strategies for improving clinical outcomes in muscular dystrophy.
